# Dentin to dentin adhesion using combinations of resin cements and adhesives from different manufacturers – a novel approach

**DOI:** 10.1080/26415275.2020.1793677

**Published:** 2020-07-16

**Authors:** Elke Seitz, Carl Hjortsjö, Jon E. Dahl, Erik Saxegaard

**Affiliations:** a Institute of Clinical Odontology, University of Oslo, Oslo, Norway; b NIOM – Nordic Institute of Dental Materials, Oslo, Norway

**Keywords:** Shear bond strength, dentin bonding, resin cement, adhesive, universal adhesive, fracture strength

## Abstract

**Aims:**

The aims of this study were to present a novel method to analyse dentin bond strength and to evaluate the bond strength of combining adhesive systems and resin cement from different manufacturers.

**Methods:**

Human wisdom teeth were ground flat to the dentin on parallel surfaces and axially cut into two parts. Dentin cylinders (Ø 3 mm) were drilled from one half of each tooth. The other half from each tooth was embedded in epoxy resin with the dentin surface exposed. The specimens were ground with silicone carbide paper and the dentin cylinders were cemented onto the dentin surface of the other half of the same tooth.

**Materials:** Resin cement and adhesive systems from three different manufacturers were used in various combinations (*n* = 8 per group). Cement and adhesive from the same manufacturer served as control. Shear bond strength (SBS) was measured and fracture modes were registered.

**Results and conclusions:** The highest median SBS value was found in a bonding combination between cement and a non-corresponding adhesive (33.1 MPa) and one of the lowest values was found in one of the controls (15.3 MPa). Cohesive fractures were most frequent. The results indicated that combining adhesive and cement from different manufacturers did not compromise the dentin bonding. The novel test method is recommended for evaluating dentin bonding.

## Introduction

Patients’ demand for highly aesthetic restorations has increased over the last decades thus promoting all-ceramic restorations, which require cementing with resin bonding systems [1]. Numerous types of luting systems are available on the market [2].

It is not unusual in clinical use to mix and interchange resin cement and adhesive systems from different manufacturers. This could be due to practical or economical issues. However, common recommendations from manufacturers of dental materials are to use their own products if products are to be combined [3–5]. One study even strictly discourages interchanging products of different manufacturers [6]. Notably, most bond strength studies use adhesives and composites or luting systems from the same manufacturers, assuming that these products are optimally tuned to each other [7]. However, some studies have combined cement and adhesives from different manufacturers without comparing the bond strength results of cementation using products from the same manufacturer [8,9].

In many studies investigating bond strength, various restorative materials are cemented to dentin substrate [2,10–14]. In these studies, the measured bond strength reveals the weakest bond which could be either to dentin or to the restorative material. We preferred to test bonding to dentin only, thus eliminating factors related to restorative materials such as composite, ceramic, primers and silanes.

The aim of the present study was first to present a new bond test method. This method evaluates the bond strength of resin-based cement to dentin only. Secondly, the study investigates the effects of combining cement and adhesive systems from different manufacturers on bond strength and compares the results to experiments using products from the same manufacturer.

The null hypothesis to be tested was that there is no difference in bond strength between specimens cemented with resin cement and adhesives from the same manufacturers and those cemented by combining cement and adhesives from different manufacturers.

## Materials and methods

### Cements and adhesives

Three different dual-cure resin cement (A, B, C) and adhesive systems (a, b, c) were tested in combinations with each other. Corresponding cement and adhesives served as controls (*).

The tested cement and adhesives with the respective cementing and adhesive procedures are displayed in Table 1.

**Table 1.** Products and cementation procedures.

Manufacturer	Code	Etchant (batch nr.) + adhesive (batch nr.)	Clinical procedure etchant/adhesive	Code	Cement (batch nr.)	Clinical procedure cementation
3M ESPE, St. Paul, U.S.A.	a	Universal Etchant *(4077339)*Scotchbond Universal Adhesive *(812058)*	15 s etch, 15 s rinse, 3 s air-dry 20 s brushing, 5 s slightly air-dry, 10 s light curing.	A	RelyX Ultimate *((01)0403507701937)*	10 N press for 10 s 20 s x 4 sides light curing
Ivoclar-Vivadent, Schaan, Liechtenstein	b	Total Etch *(X38777)*Adhese Universal Viva Pen *(W09448)*	15 s etch, 5 s rinse, 5 s air-dry thoroughly 20 s brushing, 5 s air-dry, 10 s light curing.	B	Variolink Esthetic *(W39194)*	10 N press for 10 s 20 s x 4 sides light curing
Bisco, Schaumburg, U.S.A.	c	Select HV Etch *(1800004294)* All-Bond Universal *(1800003174)*	15 s etch, 15 s rinse, slightly drying /cotton-paper 2 × 15 s brushing, 10 s air-dry, 10 s light curing.	C	eCement *(1800001560)*	10 N press for 10 s 20 s x 4 sides light curing

### Tooth collection

The study protocol was accepted by the Regional Ethical Committee for Medical Research in Norway (Reference number 2018/1996). Caries free extracted human wisdom teeth were collected after donor consent and stored in 0.1% thymol in moist cotton at 4 °C for no more than 3 months.

### Specimen preparation

**Figure 1.**
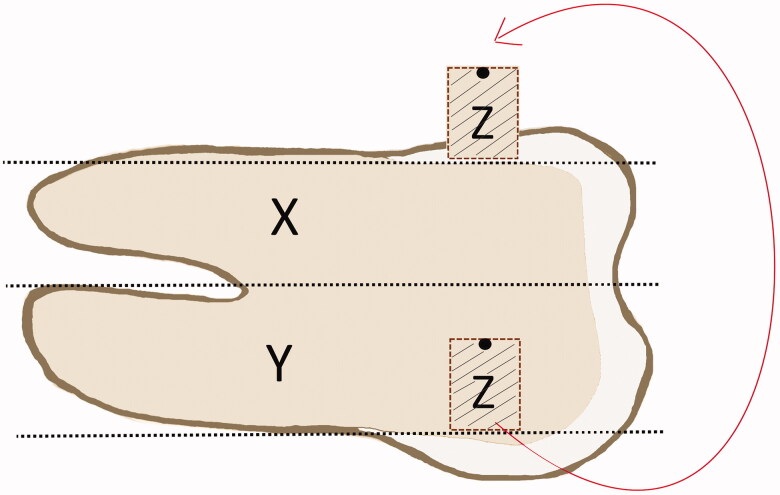
Schematic drawing of tooth and dentin specimens: axial cut giving sections X, and Y, the area for collecting the dentin cylinder specimen Z, and how the specimen Z was orientated and placed on section X. The non-test side of specimen Z was marked with black permanent marker for orientation.

The tooth crown and root were ground flat on opposite surfaces (mesial and distal or buccal and lingual) through the enamel to the dentin (Trimmer Renfert Infinity MT3/MT3pro, Renfert, Hitzingen, Germany). The teeth were axially cut into two sections, labelled “X” and “Y” (Figure 1) with a silicon carbide separating disc (Moyco Industries, Inc., Philadelphia, PA).

Section X was embedded with the dentin surfaces facing up in epoxy resin (EpoFix Resin, Struers ApS, Ballerup, Denmark) in polyethylene mounting cups (SeriForm, Struers Aps, Ballerup, Denmark). The epoxy resin cured overnight (>12 h) in an exicator at 23 °C.

The epoxy resin blocks containing section X, were mounted in a grinding machine (Phoenix 4000, Wirtz Buehler Ltd., Lake Bluff, IL) and the tooth surface was wet ground (Silicon Carbide Paper #500, Struers ApS, Ballerup, Denmark).

Section Y was embedded in cold cured acrylic resin (Paladur, Kulzer GmbH, Hanau, Germany) with the exposed plane dentin surfaces facing up and immersed in water at 40 °C (±5) for 15 min.

**Figure 2.**
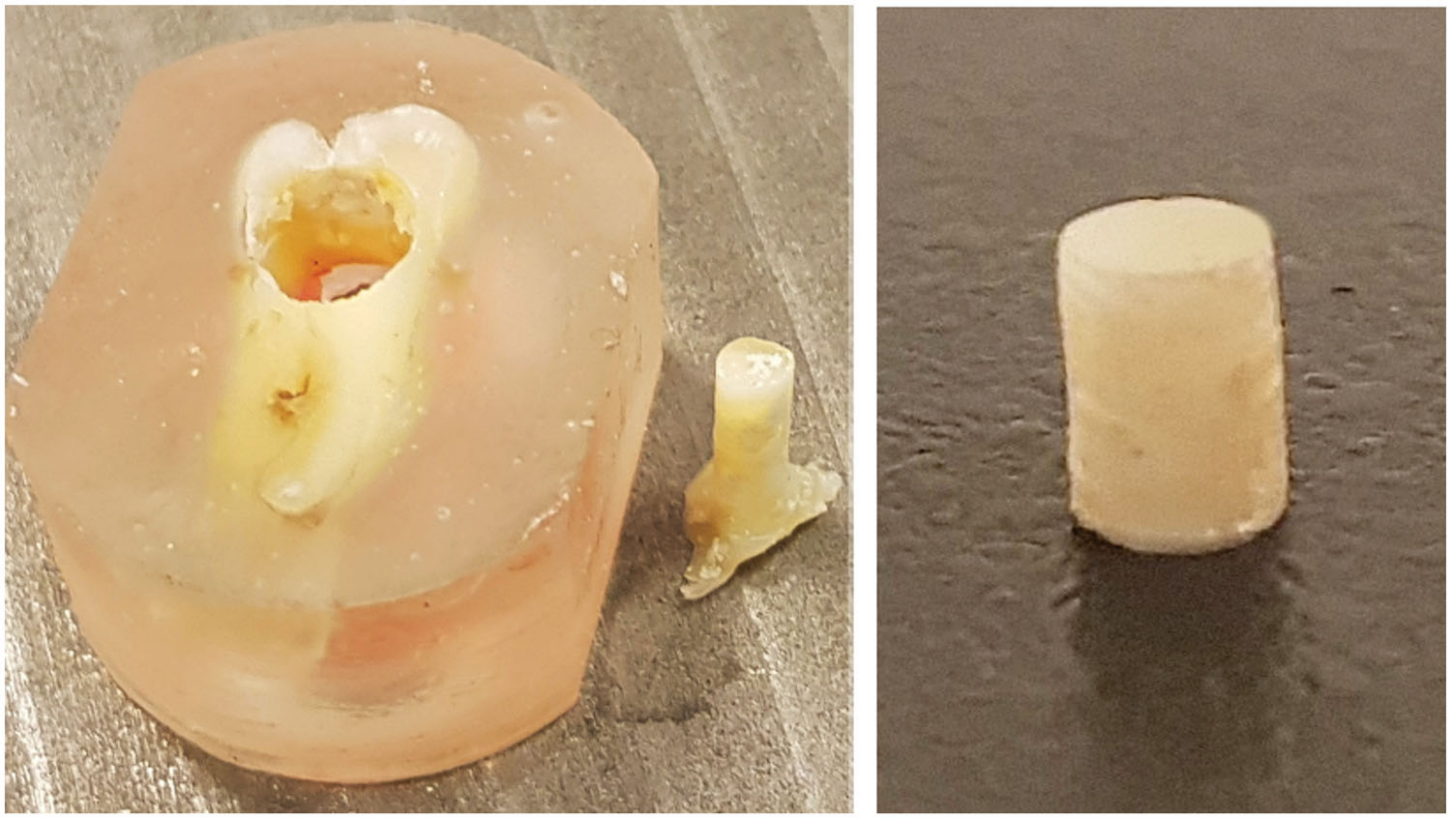
Picture of one acrylic block with the drilled dentine cylinder Z on the left side. On the right side the same cylinder after plane grinding.

The acrylic blocks containing section Y were fixed in a device assuring horizontal alignment of the dentin surfaces. Dentin cylinders labelled “Z” of approximately 3 mm height were then drilled out (Ibarmia TL-25, Ibarmia Innovatek, Azkoitia, Spain) at 850 rpm (Figure 2). The drills used were diamond-coated hollow drills (Strong drills, MegaFlis, Slependen, Norway) with inner and outer diameters of 3 and 6 mm, respectively. To ensure the equal size of the bonding surfaces of the specimens Z, the cross-sectional diameters for each cylinder were measured at three different positions (approx. 0–180°, 60–240°, 120–300°) with a digital micrometer (Digimatic micrometer series 293, 0–1′′, Mitutoyo, Kawasaki, Japan). The mean diameter of specimens Z was calculated and specimens presenting a mean diameter of 2.798–3.023 mm were accepted.

Specimens Z were mounted in a custom device and both ends of the specimens were ground with SiC grinding paper (Silicone carbide grinding paper, #500, Struers ApS, Ballerup, Denmark) to ensure that the ends were flat and perpendicular to the sample axis (Figure 2).

The surface is the originally most superficial site of the cylinder was used as the test surface (Figure 1). The specimens Z were stored in distilled water for 12–18 h at room temperature before testing.

### Cementation

**Figure 3.**
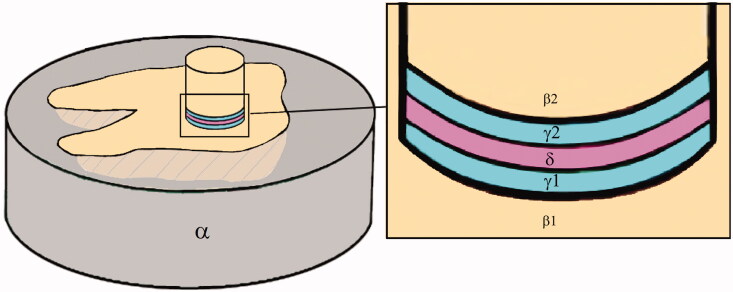
Schematic drawing of test setup and bonded components: epoxy (α), embedded section X (β1), dentin cylinder specimen Z (β2), adhesive layers (γ1, γ2) and resin cement (δ).

The dentin surfaces of sections X and specimens Z were rinsed with water, dried, and etched with the respective phosphoric acids corresponding to the adhesive systems and rinsed with water and dried (Figure 3).

Holes (Ø 3 mm) were punched out of black sticky tape pieces before attaching the tape onto the dentin surfaces of sections X in order to expose the dentin area intended for cementation only [7,15,16]. Adhesive material was applied with a micro brush (Quick-Stick, Dentsolv, Saltsjo-Boo, Sweden) on specimens Z and sections X and light-cured for 10 s (Mini L.E.D., Acteon Satelec, 1250 mW/cm^2^, Merignac, France). The bonding and cementation procedures were carried out according to the manufacturers’ instructions (Table 1).

Resin cement was equally applied in section X and specimen Z with the micro brush. Each specimen Z was positioned vertically in the restricted contact area on the corresponding section X and a vertical load of 10 N was applied for 10 s. Excess cement was removed with a micro brush and the specimen was preliminary light-cured for 3 s. Then the tape was carefully removed with the aid of a scalpel and the light-curing finalized from 4 directions, each of 20 s, and stored in water at 37 ± 2 °C for 24 h prior to testing.

### Shear bond strength (SBS) test

The test specimen was mounted in the testing apparatus (LRX Series Materials Testing Machine, NS-EN ISO 7500-1, AMETEK, Lloyd instruments, Hampshire, UK) and SBS test was performed with the speed of 1.0 mm/min until failure.

The maximum force (N) at failure was recorded (Nexygen MT Material Testing Software Version 4.5., AMETEK, Lloyd Instruments, Hampshire, UK) and the bond strength was calculated in MPa (force/bonded area) according to modified ISO standards 11405:2015(E) [15] and 29022:2013 [16].

The surfaces of section X and specimen Z were examined with a light stereo microscope, 20–25× magnification (Euromex Nexius Zoom EVO, Euromex, Arnhem, Netherlands) to determine the fracture mode. The fracture mode was categorized into three types:

1. “adhesive” at the cement/bonding interface (≥70% of the total area not covered by the cement of both fracture surfaces added together).
2. “cohesive” in cement (≥60% of each of the surfaces covered by cement).
3. “mixed” (those which did not fall into groups 1 or 2).

In addition, the surfaces of two of the specimens were examined using a scanning electron microscope (SEM) with element analysis to detect if the dentin surfaces were covered with adhesive or cement remnants (TM4000Plus Scanning Electron Microscope with Energy Dispersive X-Ray Spectrometers, Hitachi High-Technologies Corporation, Tokyo, Japan).

All tests were done as strictly as possible according to ISO standards [15,16] and with the same operators and the same room conditions. The manufacturers’ advice was followed correctly. To imitate blinding in the present study, the cement and adhesives were used randomly.

### Statistical analysis

SBS test data and fracture modes were analysed using SPSS Version 25 (IBM, New York, US) and Microsoft Excel (Office version 365, Microsoft Corporation, Washington, US). If Kolmogorov–Smirnov test failed, non-parametric methods were used for statistical analysis, Kruskal–Wallis ANOVA and Mann–Whitney test to detect the median differences between the combinations. The effect size was calculated with Eta square η^2^. Descriptive statistics and Fisher’s Exact Test were used to analyse any statistical connections between fracture modes and combinations. Statistical significance was set at the .05 level.

## Results

**Figure 4.**
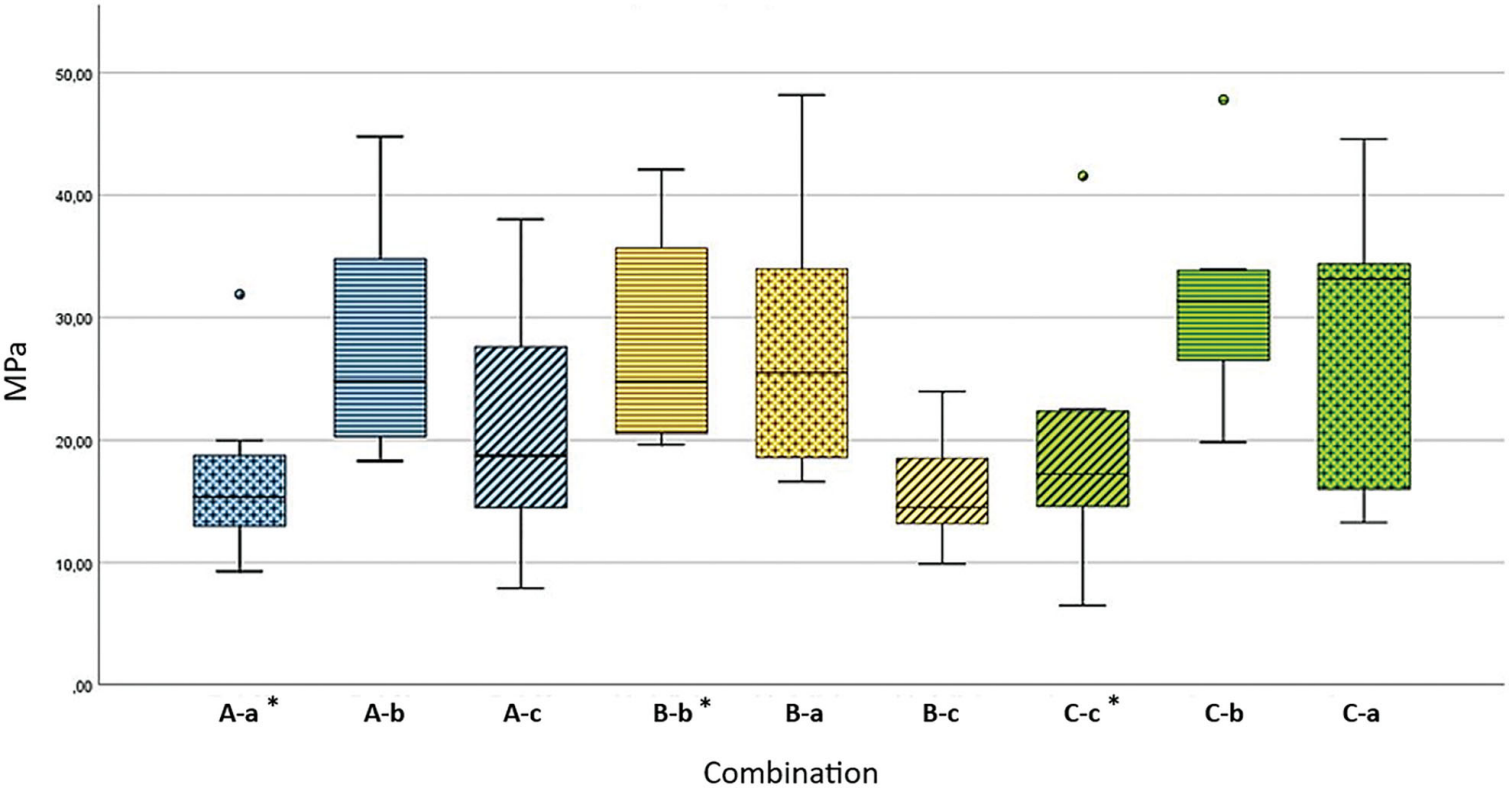
Comparison of all 72 samples (9 combinations, *n* = 8 specimens per combination). Boxplot of MPa values by combinations: median, maximum, minimum, 25 interquartile range, 75 interquartile range, *control group.

The results of the SBS tests are described in Figure 4 and Table 2. Statistical analysis (Kruskal–Wallis ANOVA) of the overall data showed significant differences between the resin cement/adhesive combinations (*p* < .05) (Figure 4).

**Table 2.** Comparison 2 by 2 of the combinations (each *n* = 8) with the non-parametric Mann-Whitney test.

Cement-adhesive combination	MPa mean (SD)	Cement-adhesive combination	MPa mean (SD)	*p*-Value	eta square *η*^2^ effect size
C-a	28.1 (11.4)	B-c	15.8 (4.5)	.036	.294
C-b	31.4 (8.3)	A-c	20.9 (9.7)	.036	.294
C-b	31.4 (8.3)	C-c*	19.5 (10.3)	.016	.389
C-b	31.4 (8.3)	A-a*	16.9 (6.9)	.005	.536
C-b	31.4 (8.3)	B-c	15.8 (4.5)	.002	.618
B-a	27.6 (10.9)	A-a*	16.9 (6.9)	.016	.388
B-a	27.6 (10.9)	B-c	15.8 (4.5)	.009	.175
A-b	27.8 (10.0)	A-a*	16.9 (6.9)	.012	.424
A-b	27.8 (10.0)	B-c	15.8 (4.5)	.006	.497
B-b*	28.0 (9.3)	A-a*	16.9 (6.9)	.012	.424
B-b*	28.0 (9.3)	B-c	15.8 (4.5)	.006	.497

Statistically significant differences were found in the displayed pairs of combination groups. *control group. Displayed the SBS results with MPa values: mean and standard deviation (SD). Significance level is set to *p* ≤ .05. Eta-square showed in all samples a large effect size (*η*^2^ > .14). In all the other combinations there were no statistical differences found.

Statistically significant differences (Mann–Whitney) between the combinations are shown in Table 2.

**Figure 5.**
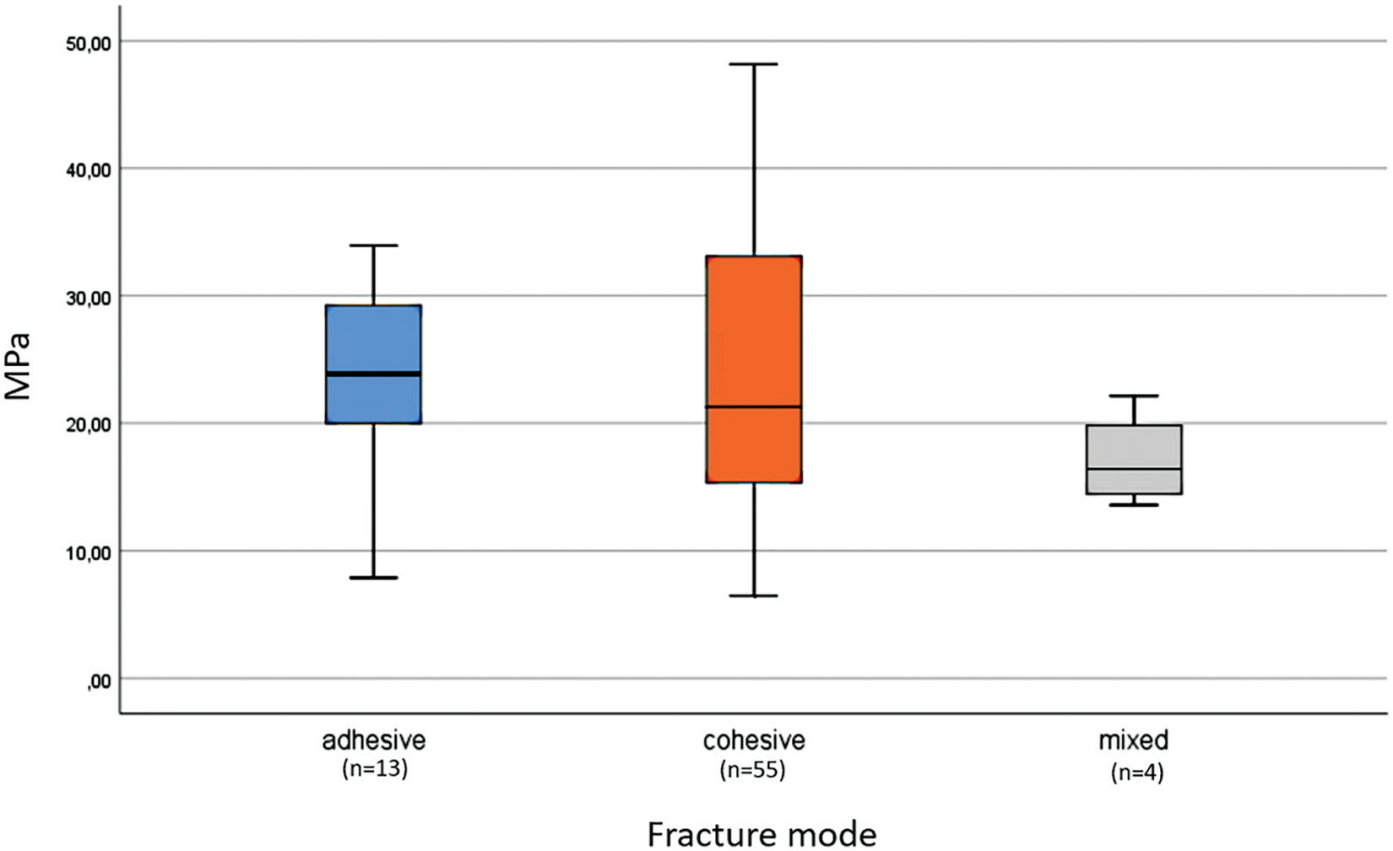
Boxplot of MPa values and fracture mode for all samples. Fracture mode analyses showed 18% adhesive fractures, 76% cohesive fractures and 6% mixed fractures.

**Figure 6.**
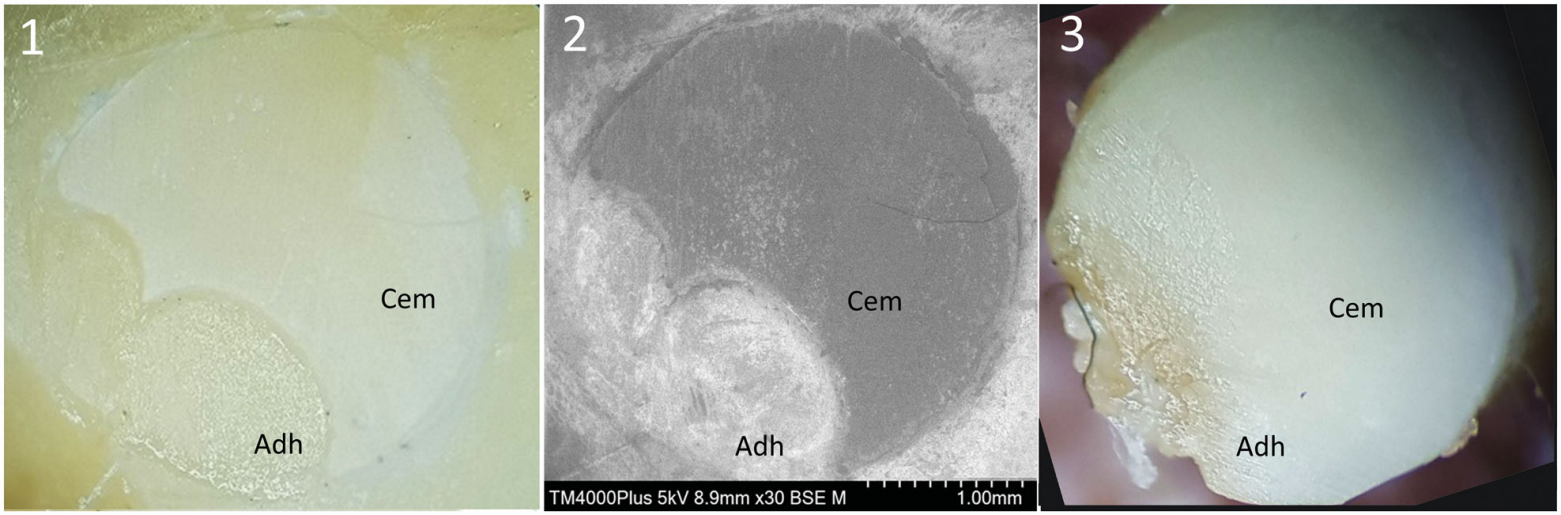
Examples of a mostly cohesive fracture morphology: Combination B-c. (1) Tooth surface X (light microscope), (2) tooth surface X (SEM), (3) dentin cylinder, surface Z (light microscope). Surface Adh: adhesive, Cem: resin cement.

There were 76% cohesive fractures, 18% adhesive fractures and 6% mixed fractures. A statistically significant correlation was neither found between fracture mode and bond strength, nor between fracture mode and resin cement/adhesive combinations (Fisher’s Exact Test). The highest number of cohesive fractures were seen in the test specimen having bond values of 10–19 MPa, the highest number of adhesive fractures for the values 20–29 MPa (Figures 5 and 6). Adhesive fractures could be found in the combinations A-a*, A-b, A-c, B-a, C-a, and C-b.

Additional SEM examination of two specimens supported the results from the light microscopic examination of fracture morphology. Dentin fractures were not observed in any of the samples.

## Discussion

This novel test method using two human dentin specimens from the same tooth may be a suitable method to study dentin bonding without introducing factors related to the bonding to a restorative material. We could not find any other studies using a similar test method as in this study. The purpose of the novel developed method, only cementing dentin specimens together, was to limit the number of variables in the SBS test [17] and thereby avoiding confounding effects. The only components were dentin, adhesive system, and cement. Other material factors, for example, bond strength to ceramic specimen, ceramic primer characteristics or surface treatment of the specimen as sandblasting, which could distort the results, were thus eliminated. The tests were carried out in accordance with ISO standards 11405 and 29022 [15,16]. The aim was to measure the bond strength values in the SBS test and to assess the differences between various combinations of adhesive systems and resin cement. There is no ISO standard that only uses dentin-to-dentin as a test method. Tooth substrate, adhesive, cement and restorative materials are always included in the tests. Only testing the pure adhesion of resin material to dentin is a new and reliable aspect of SBS tests.

The two dentin specimens cemented together were obtained from the same tooth. By carefully grinding off the enamel, we got a consistent superficial dentin substrate for all specimens, avoiding undesirable effects of regional differences, for example, peripheral dentin vs. central dentin or different dentin tubule orientation as well as differences among dentin structure taken from different teeth [10,13,18]. The method to obtain two dentin specimens from one tooth also reduced the required number of human teeth. The surface morphology of the dentin specimens may influence the bond strength. Preparing with SiC paper may not be ideal as it can be argued that dentin is best prepared by a bur in order to simulate clinical practice [19]. However, this would be more difficult to standardize for trials. The roughness of the wet grinding paper used in this study was about 30 µm according to the Federation of European Producers of Abrasives corresponding approximately to the roughness of an extra-fine dental diamond bur [20].

Dentin bonding is accompanied by complicated chemical interfacial interactions between the adhesives and the bonded substrate [21]. In the present study, the dentin surface was acid-etched followed by application of the mild universal adhesives Scotchbond Universal pH 2.7, Adhese Universal pH 2.5–3, or the ultra-mild All-Bond Universal pH > 3. Ultra-mild and mild universal adhesives showed an improved bond strength to dentin when used with etch-and-rinse approach [22]. H_3_PO_4_-etching for three seconds improved the interaction depth of the tested universal adhesive without overexposing demineralized collagen or reducing Ca-content availability at the bonded interface. Acid etch might increase the dentin bonding effectiveness of universal adhesives and the resistance to water aging [23]. These studies confirm that our test setup to etch dentin prior to bonding is acceptable. In this study the load of 10 N before curing resulted in a uniform minimal thickness of the cement layer for all specimens. The cement film thickness is often discussed as an important factor in aging and fatigue resistance. May et al. [24] found that failure loads of bonded CAD/CAM crowns depended on the bonding condition and the cement thickness with at least double fracture load at 50 µm cement thickness than at 500 µm. Another study found that trilayer glass-ceramic/cement/composite specimens with thick resin-cement layers stored for 60 days in water presented significantly lower reliability under fatigue testing. The high reliability found for thin cement layers was not lowered with water aging [25].

The term “Universal” reflects manufacturers’ claims that these adhesives can be applied with any adhesion strategy and offer the versatility of use with a variety of direct and indirect restorative materials [11]. It implicates that the use of universal resin cement or adhesives should be fully compatible and combinable with other methacrylate resins, composites, and adhesives. Paradoxically, many dental companies promote their resin cement in combination with their own adhesive materials exclusively, while at the same time in their instructions for use advocate their adhesives to be compatible with all other methacrylate-based materials.

To our knowledge, no other studies have compared combinations of adhesives and resin cement from different manufacturers. As noted by Van Meerbeek et al. [7] it is surprising that adhesives were usually tested along with the respective composites of the same company in the sense that the materials were fine-tuned to each other. Conclusions of comparative bond strength tests can only be drawn at the level of the used combinations and not at the level of the adhesive itself [7,17]. Adhesion testing with SBS is a technique sensitive procedure and the biological dentin substrates employed are not standardisable [17].

There were statistically significant differences in MPa values in our test. The non-corresponding combination C-a showed the highest SBS values followed by C-b. The lowest values were measured with the non-corresponding combination B-c and the corresponding combination A-a. These results lead to the conclusion that the cement and adhesives of different manufacturers can be combined without compromising the bond strength. Because of the significance and the strong effect size as shown in Figure 4, we can assume that our test results can be transferred to clinical practice. However, there is still a lack of correlation of the absolute value of bond strength with the clinical performance [7,12]. The reason could be that the absolute value of bond strength above a certain threshold value is irrelevant for the clinical performance [12]. In addition, bond strength methods usually apply force in one direction (transverse or longitudinal) in contrast to the clinical situation with multidirectional forces.

We have tested dentin to dentin bonding and the test values cannot directly be compared to studies using a more complex setup including dentin, cement, and restorative material. Values both above and below ours have been reported [2,10,12]. In such complex setups, the bond strength values represent only the weakest link of the adhesion chain which could be that to the restorative material. The bond strength values in our study show some variation. It could be found quite large dispersions and a large standard deviation also in other bond strength studies using dentin/universal adhesive/ceramic specimen [9].

There is no consistency in the literature on how to define adhesive/cohesive and mixed fractures. According to ISO standards for bond measurements, the fracture modes could help to explain the results [15,16]. However, no clear definition of the various fracture modes is given [15,16]. In the present study, the fracture modes were defined post-hoc as we could not find cohesive fractures in dentin and as all surfaces were found to be covered by adhesive and/or cement. An adhesive fracture between adhesive and cement was therefore defined as ≥70% of the total area not covered by the cement of both fracture surfaces. On the other side, we defined a cohesive fracture model in cement when ≥60% of each of the surfaces > covered by cement. All other fractures were defined as mixed. With this test method, the number of interfaces was reduced. The dentin/adhesive interfaces (β1/γ1 and β2/γ2) were defined as one interface and the cement/adhesive interfaces (γ1/δ/γ2) were also defined as one interface as they were of the same material (Figure 3).

Figure 6 shows a typical cohesive failure where the fracture occurs primarily within the cement layer and not at the adhesive/cement interface. Our test results confirm that the adhesion between adhesive and cement is stronger than the cement itself. In this study, most of the specimens (76.4%) showed cohesive fractures with cement remnants on both surfaces X and Z (Figure 5). Adhesive fractures at the resin/cement interface were seen in 18.1% and mixed fracture modes in 5.6%. Adhesive fractures could be found in 6 of the 9 combinations. The highest number of adhesive fractures were found in the group 20–29 MPa. The highest number of cohesive fractures were seen in the group 10–19 MPa, (Figures 5 and 6). There was no statistically significant correlation between fracture mode and bond strength values nor between fracture mode and resin cement/adhesive combinations (A, B, C/a, b, c).

To verify the light microscopic fracture mode analyses, we did an additional surface examination of two of the samples using SEM with element analysis. This confirmed our results of the light microscopic examination. The SEM topographical and element analysis showed that the dentin surfaces were covered by remnants of adhesive or bonding, thereby substantiating that there was no cohesive fracture in the dentin and no adhesive fracture at the dentin/adhesive interface.

Liebermann et al. [9] conducted a tensile bond strength test study to test the impact of different universal adhesives in combination with one resin cement and different CAD/CAM ceramics. It seems difficult to evaluate the influence of the adhesives only and to exclude the influence of ceramics, ceramic primers or silanes. In contrast to the study of Liebermann et al. [9] we could not find a statistically significant correlation between the bond strength values and the fracture modes. There was no significant correlation found between the fracture morphology and the bonding combinations. But in the group with the highest bond strength values, there were no adhesive fractures. Mainly cohesive or mixed fractures show that the bonding between the adhesive and the tooth is stronger than the resin cement layer itself. This gives reason to assume, that the resin cement seem to be the weakest link in the cement adhesive combinations as suggested in other recent studies [14] and that the strongest parts are the adhesives.

The present study investigated the bond strength of mixed combinations of universal adhesives and resin cement of different manufacturers with a novel test method. According to our test results, the recommended corresponding combinations did not show the strongest bonding values in the SBS test. The null hypothesis was rejected (*p* < .05). The results of our study suggest that it is possible to combine products from different dental companies. However, this statement is based on short-term results. Long-term storage and thermal cycling may alter the results and need to be investigated. Another limitation of this study is the low sample size. The overall responsibility for the use of dental materials is always on the shoulders of the clinicians, thus it is recommendable to follow the manufacturers’ advices. It would be desirable though, both for the clinician and the patient, if dental companies were more transparent and open to accepting combinations of their own adhesive materials with those of their competitors.

## Conclusions

Based on the results and within the limitations of this laboratory study, it can be concluded that

- Using alternating combinations of resin cement and adhesives seem possible.
- The novel test method using two human dentin specimens from the same tooth proved useable in SBS-testing of resin cement-adhesive combinations.
- The manufacturers’ corresponding resin cement-adhesive combinations did not show the strongest bonding values in the SBS test.
- The resin cement were the weakest components in the bonded dentin-adhesive-cement-complex.
